# Lung adenocarcinoma mimicking pulmonary fibrosis-a case report

**DOI:** 10.1186/s12885-016-2763-6

**Published:** 2016-09-13

**Authors:** Bakir Mehić, Lina Duranović Rayan, Nurija Bilalović, Danina Dohranović Tafro, Ilijaz Pilav

**Affiliations:** 1 Clinic of Pulmonary Diseases and TB, University Clinical Centre of Sarajevo, Bardakčije 90, 71000 Sarajevo, Bosnia and Herzegovina; 2 Clinical Pathology and Cytology, University Clinical Centre of Sarajevo, Bolnička 25, 71000 Sarajevo, Bosnia and Herzegovina; 3 Clinic for Thoracic Surgery, University Clinical Centre of Sarajevo, Bolnička 25, 71000 Sarajevo, Bosnia and Herzegovina

**Keywords:** Lung adenocarcinoma, Pulmonary fibrosis, Diagnosis, Interstitial opacities, Progressive dyspnea, Case report

## Abstract

**Background:**

Lung cancer is usually presented with cough, dyspnea, pain and weight loss, which is overlapping with symptoms of other lung diseases such as pulmonary fibrosis. Pulmonary fibrosis shows characteristic reticular and nodular pattern, while lung cancers are mostly presented with infiltrative mass, thick-walled cavitations or a solitary nodule with spiculated borders. If the diagnosis is established based on clinical symptoms and CT findings, it would be a misapprehension.

**Case presentation:**

We report a case of lung adenocarcinoma whose symptoms as well as clinical images overlapped strongly with pulmonary fibrosis. The patient’s non-productive cough, progressive dyspnea, restrictive pattern of pulmonary function test and CT scans (showing reticular interstitial opacities) were all indicative of pulmonary fibrosis. The patient underwent a treatment consisting of corticosteroids and antibiotics, to no avail. Histopathology of the lung showed that the patient suffered from mucinous adenocarcinoma. Albeit the immunohistochemical staining was not consistent with lung adenocarcinoma, tumor’s morphological characteristics were consistent, and were used to make the definitive diagnosis.

**Conclusion:**

Given the fact that radiography cannot always make a clear-cut difference between pulmonary fibrosis and lung adenocarcinomas, and that clinical symptoms often overlap, histological examination should be considered as gold standard for diagnosis of lung adenocarcinoma.

## Background

Adenocarcinoma of the lung is the most common type of lung cancer, accounting for approximately one half of all lung cancer cases. The increased incidence of adenocarcinoma is thought to be due to the introduction of low-tar filter cigarettes in the 1960s, although this causality has never been confirmed [1]. Histological diagnosis requires evidence of either neoplastic gland formation or intracytoplasmic mucin. There are significant variations in the extent and architecture of neoplastic gland formation, ranging from well-formed acini to more papillary and even cribriform types [2]. Lung adenocarcinoma, especially types with lepidic growth pattern, can have highly variable clinical presentation, and range from a small solitary nodule or limited number of nodules, to more extensive miliary disease, or diffuse parenchymal infiltrates that are similar in appearance to bacterial pneumonia [3, 4] Because of these characteristics, it’s often called “masquerader“ [3, 5]. The exact mechanism of lung adenocarcinoma pathogenesis is still being investigated, however it appears that tumor proliferation is eclipsed by noticeable inflammation and fibrosis that mimic a benign inflammation [5], thus confusing physicians and consequently delaying diagnosis, as well as affecting quality of patients’ life. Traditionally, classification of lung carcinoma has been based solely on evaluation of routinely stained biopsies or cytological smears. However, ancillary tests such as immunohistochemistry are being increasingly used to aid pathologists in diagnosis of subtypes. Lung adenocarcinoma shows specific staining patterns, which are useful in the differential diagnosis of poorly differentiated neoplasms. The following patterns are positive: TTF-1, napsin A, CK 7, mucicarmine, PAS-D [6, 7]. In 2011, a multidisciplinary expert panel representing the International Association for the Study of Lung Cancer (IASLC), the American Thoracic Society (ATS), and the European Respiratory Society (ERS) proposed a major revision of the classification system. These changes primarily affect the classification of adenocarcinoma and its distinction from squamous cell carcinoma. The 2011 IASLC/ATS/ERS classification of lung adenocarcinoma schema stresses the importance of radiographic findings in this approach to classification of lung adenocarcinoma [8].

Here we report a case of lung adenocarcinoma that mimicked pulmonary fibrosis, as based on the clinical symptoms and radiographic images. Also, in this case the diagnosis of mucinous adenocarcinoma of pulmonary origin was determined based on histological type, despite the unusual immunohistochemical pattern for this type of lung cancer.

## Case presentation

**Fig. 1:**
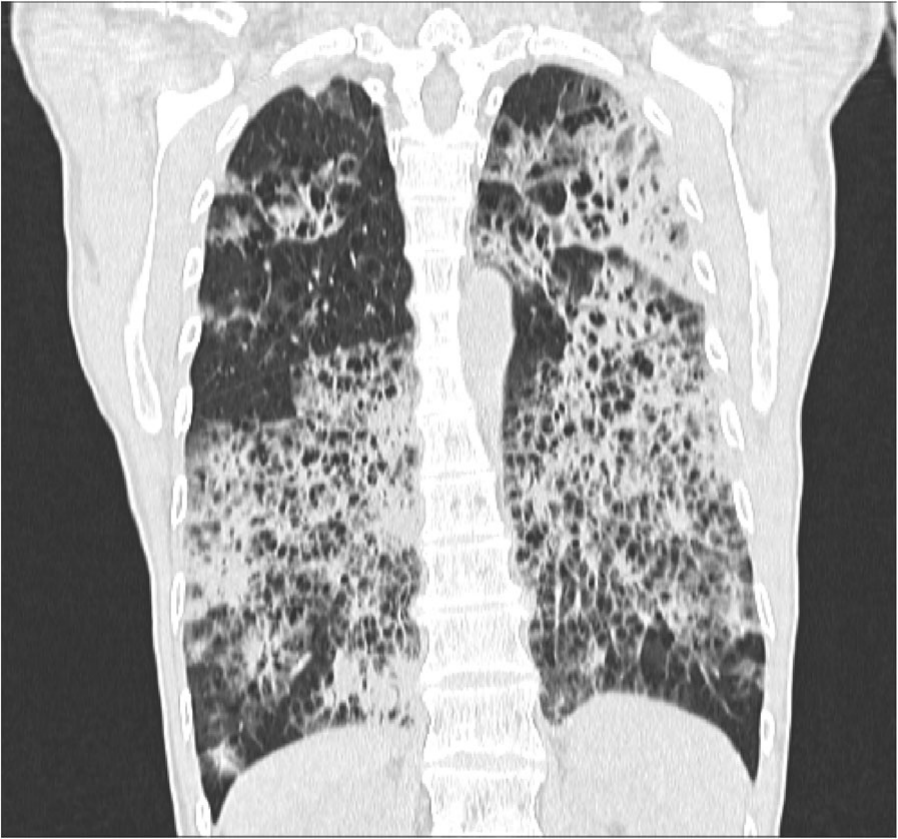
Computed tomography scan of the thorax with coronal view demonstrating of reticular interstitial opacities

**Fig. 2:**
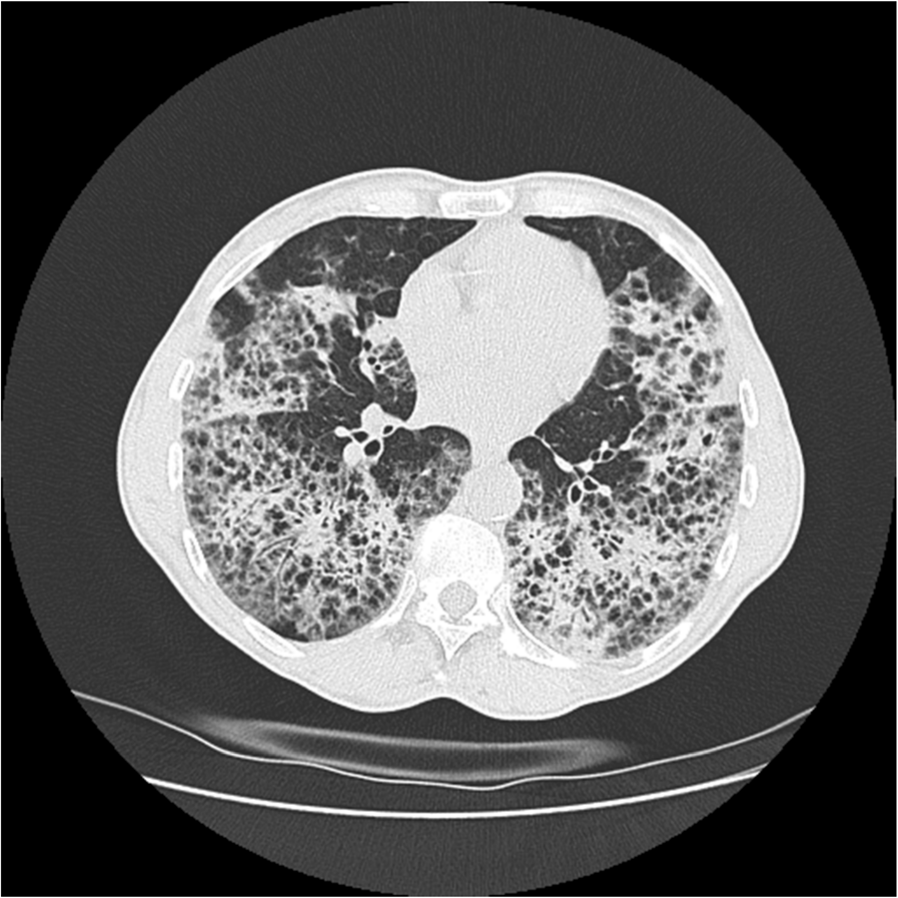
Computed tomography scan of the thorax with axial view demonstrating of reticular interstitial opacities with plenty of thick mucus, and thickening of interlobular septa

A 59 year-old woman, smoker 22 pac/year, presented to hospital with 4 months of worsening dyspnea, nonproductive cough at first, but lately she was able to cough up a thick, white sputum. She has lost 5 k since the beginning of the disease. She denied having hemoptisis and systemic infection symptoms, such as fever, chills and sweats. The patient’ had been regularly taking therapy for high blood pressure for 10 years. Pulmonary examination showed notably descended and immovable hemidiaphragms with decreased breath sounds accompanied by some low-pitched whistles. Lung function testing registered medium level restrictive disturbances of ventilatory insufficiency. DLCO was 27 %. Gas analysis of arterial blood registered hypoxic respiratory failure (PaO_2_ 7.14 PaCO_2_ 4.61 pH 3.37 SaO_2_ 83 %). Chest X-ray demonstrated bilateral reticular opacities with honeycombing with predominant sub pleural distribution. CT confirmed findings of reticular interstitial opacities with extended and deformed small airways filled with plenty of thick mucus, visible bronchiectasis and thickening of interlobular septa (Figs. 1 and 2). Bronchoscope findings were unremarkable with a lot of mucus gushing from the segmental bronchi. Histopathological finding of transbronchial biopsy as well as cytological examinations of bronchoaspirate was not conclusive. Progressive course of the disease without response to antibiotics and corticosteroid therapy indicated underlying malignant disease.

**Fig. 3:**
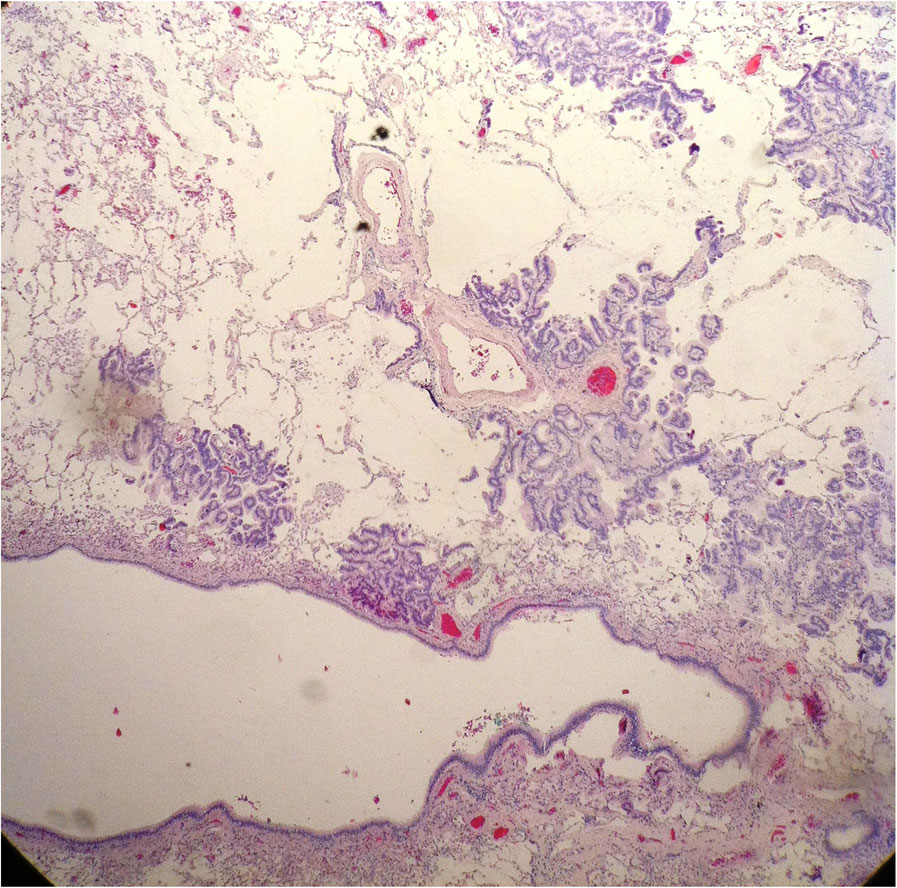
Tissue histology revealed mucinous adenocarcinoma of the lung whose pattern was largely lepidic with smaller foci acinar growth

**Fig. 4:**
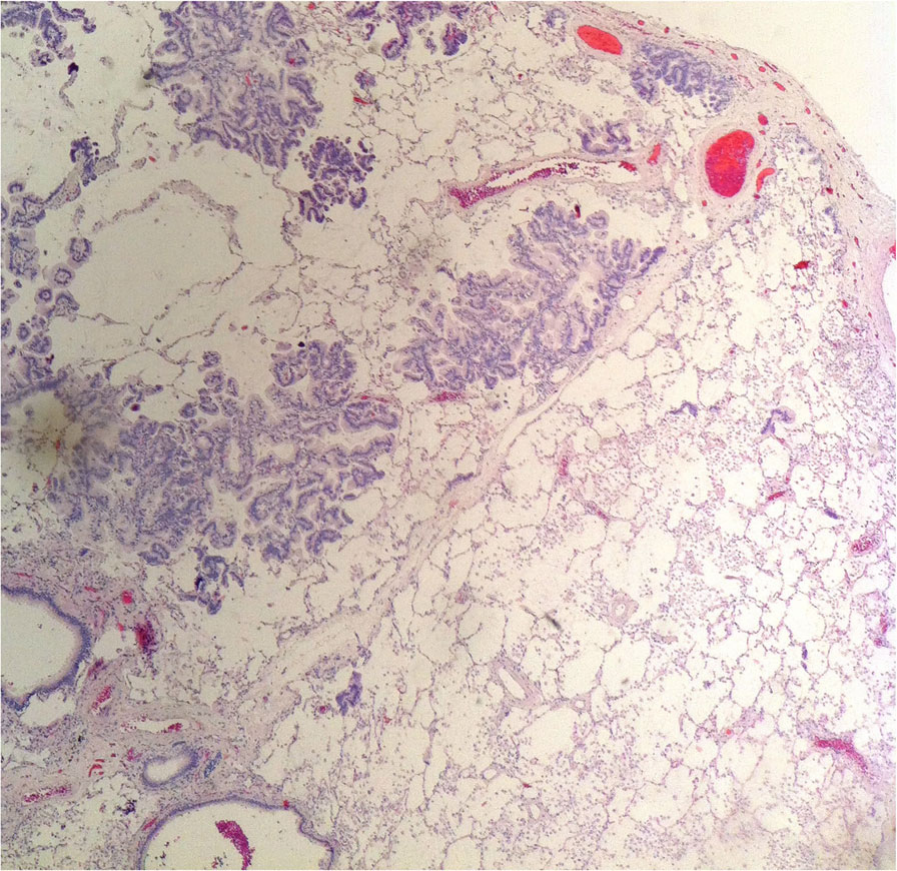
Close-up view of mucinous adenocarcinoma of the lung whose pattern was largely lepidic with smaller foci acinar growth

Consequently the patient underwent video-assisted thoracoscopy with lung biopsy under general anesthesia. Tissue histology revealed mucinous adenocarcinoma of the lung with pleural infiltration (stage PL1); pattern was largely lepidic with smaller foci of acinar growth (Figs. 3 and 4), partially thickened septa and several cuts presented expanded air pathway that creates the image of centriacinar emphysema. Immunohistochemical finding was TTF1 negative, napsin negative, CDX2 negative, CK7 positive, and CK20 negative. In addition, approximately 20 % of lung adenocarcinomas are TTF1 negative, which further complicates the diagnosis [8–10]. Despite the fact that immunohistochemical staining wasn’t specific for mucinous adenocarcinoma, histological diagnosis was determined by morphological features of the tumor.

There were no detected mutations in EGFR gene. KRAS wild type genotype was detected on codons 12 and 13. Biopsy tissue didn’t show signs of pulmonary fibrosis. Seven days later, the patient died presenting terminal respiratory failure.

## Conclusion

Given the fact that radiography cannot always make a clear-cut difference between pulmonary fibrosis and lung adenocarcinomas, and that clinical symptoms often overlap, histological examination should be considered as gold standard for diagnosis of lung adenocarcinoma.

## Abbreviations

CDX2: A highly sensitive and specific marker of adenocarcinomas of intestinal origin
CK20: Cytokeratin-20
CK7: Cytokeratin-7
CT: Computed tomography
DLCO: Carbon monoxide diffusing capacity of the lung
EGFR: Epidermal growth factor receptor
KRAS: KRAS proto-oncogene
PaCO_2_: Partial pressure of carbon dioxide
PaO_2_: Partial pressure of oxygen
PAS-D: Periodic acid–Schiff–diastase
pH: Measure of acidity or alkalinity of an aqueous solution
SaO_2_: Oxygen saturation
TTF1: Thyroid transcription factor-1
